# The Impact of Physical Activity on Weight Loss in Relation to the Pillars of Lifestyle Medicine—A Narrative Review

**DOI:** 10.3390/nu17061095

**Published:** 2025-03-20

**Authors:** Natalia Niezgoda, Tomasz Chomiuk, Przemysław Kasiak, Artur Mamcarz, Daniel Śliż

**Affiliations:** 3rd Department of Internal Medicine and Cardiology, Medical University of Warsaw, 04-749 Warsaw, Poland

**Keywords:** physical activity, obesity, weight loss, lifestyle medicine

## Abstract

Currently, overweight and obesity are key problems globally. Several modifiable factors influence weight management. The number of obese and overweight people has significantly increased over the past few decades. Therefore, it is crucial to find effective and tailored strategies for weight management in public health and medicine. It has become necessary to take a comprehensive look at the problem of obesity and the process of weight loss, taking into account various aspects of lifestyle. To date, the effectiveness of dietary interventions, training interventions, or a combination of both has been repeatedly studied, with varying results, but a combination of properly selected diet and physical activity is considered the most effective therapy. Physical activity is one of the main tools in the treatment of obesity, in part due to its direct effect on body weight by increasing energy expenditure, especially when paired with other elements of lifestyle. The effect of physical activity is broad, and to properly implement it in obesity therapy, it is necessary to understand its impact on aspects such as body composition, food intake, sleep, alcohol use, and mental state. The primary aim of this review is to present the influence of physical activity on weight loss in combination with the influence of physical activity on other pillars of lifestyle medicine in adults. The secondary aim is to present various dietary, exercise, and combined interventions on weight loss with their efficacies.

## 1. Introduction

The human organism strives to maintain equilibrium in a constantly changing environment. The changes that occur in human adaptation can be described using the concepts of homeostasis and homeodynamics. Homeostasis is the ability to maintain the relative stability of an organism’s internal environment with the help of physiological regulations that counteract possible disturbances. Homeostasis is necessary for an organism to adapt to the changing external environment [1]. It works mainly through negative feedback, inhibiting the receiver’s response with the sender’s signal [2]. Feedback can also be positive. When this occurs, the activities of two responses intensify each other, causing a significant increase in the effects of their activities and moving the system away from equilibrium. Describing the regulation of physiological activities, there is always some degree of chaos in the systems, which is a natural feature of any living and healthy organism. A reduction in the degree of chaos through a feedback deficit—for example, in disease or in the aging process of the organism—is expressed by a reduction in adaptability to environmental changes [3]. Therefore, the term homeostasis extends to homeodynamics. Homeostasis seeks to maintain constancy within a system, while homeodynamics takes into account the dynamics of human physiology and the changes that naturally occur during human life. Chaos provides vitality and dynamism, but stability and functioning within broad norms impose certain limitations [4].

Similarly, the human body strives to maintain a balance in terms of energy. Energy balance regulates energy expenditure and intake. A negative energy balance results in weight loss, while a positive energy balance promotes weight gain. However, energy balance is a dynamic process, and body weight changes over time. These changes occur because energy balance is tightly controlled by food and energy expenditure. Therefore, it is necessary to understand the components of energy balance to appreciate the effect of exercise on body weight and to determine why the two do not always have a linear relationship [5]. The components of energy balance have been described before in previous publications [6,7].

Obesity is a disease in which there is a disruption of the body’s homeostasis at the cellular and tissue levels, resulting in a significantly increased risk of many health complications [8].

The World Health Organization defines obesity as BMI > 30 kg/m^2^ and describes it as a health risk caused by abnormal or excessive fat accumulation [9]. In 2022, the worldwide prevalence of overweight was 43% and obesity was 16% [10]. The development of obesity is fostered by the obesogenic environment, which has contributed to a significant increase in the number of obese people worldwide over the past few decades. The problem is that the environment seems to bypass the homeostatic regulatory system, leading to obesity. It has been hypothesized that the obesogenic environment affects the neuronal node that is most crucial for defending body weight, thus resetting the level of defended body weight in obese individuals [11].

In addition to energy imbalance, the development of obesity is indirectly influenced by many aspects of lifestyle, especially the key ones which help prevent diabetes, cardiovascular disease or obesity, have been called the pillars of a healthy lifestyle. These include nutrition, physical activity (PA), sleep, stress management, avoidance of risky substances and social connection [12,13].

Despite advances in investigating the relationships between PA and weight loss, there is still some ambiguity on the weight loss benefits of PA when paired with the major pillars of a healthy lifestyle. Moreover, there is a need for a summary of recent findings on the improvement of general health and well-being as a result of weight loss due to PA and lifestyle modifications. Therefore, we address those issues in the present review. Specifically, this review aims to present the influence of PA on weight loss in combination with the influence of physical exercise on the other pillars of a healthy lifestyle.

## 2. Methodological Approach and Search Strategy

We selected studies published in the PubMed/MEDLINE database between 1 January 2006 and 31 December 2023. Specifically, for this narrative review we included only peer-reviewed articles on overweight or obese but otherwise healthy adults (>18 years). Moreover, each study must have lifestyle interventions (training, dietary, psychological, sleep or a combination of them). Finally, all studies must report data on the weight loss of the participants.

## 3. Effects of Physical Activity on Pillars of Healthy Lifestyle

According to the World Health Organization (WHO), PA is any body movement performed by skeletal muscles that requires energy expenditure. Exercise is a subcategory of PA that is purposeful, planned, structured and repetitive [14].

Regular PA at a moderate level contributes to improved overall health, including insulin sensitivity, improved lipid profile, blood pressure and body composition [15,16,17]. In addition, low levels of physical fitness are one of the risk factors for metabolic syndrome and overall mortality [18,19]. Reduced PA is strongly associated with an increased risk of obesity [20]. Increasing energy expenditure through exercise with reduced energy intake can help reduce excess body fat [21].

### 3.1. Physical Activity and Nutrition

As mentioned in the introduction—the imbalance between energy intake and expenditure is a major cause of overweight and obesity. Diet is key in this regard. Scientific evidence supports the implementation of dietary interventions based on healthy eating and changing eating habits permanently rather than restricting calories or individual nutrients. Dietary advice must therefore focus on the overall dietary pattern as part of the treatment of metabolic syndrome rather than a single intervention [22].

The effect of exercise on the regulation of food intake is important. Many studies have observed appetite-related responses during and after single bouts of continuous aerobic exercise. These studies indicate that subjective feelings of appetite are transiently suppressed during exercise performed at or above an intensity of 60% peak oxygen uptake or maximal heart rate [23]. This phenomenon is referred to as exercise-induced anorexia and supports weight loss. Appetite typically returns to resting control values within 30 to 60 min after exercise and does not result in changes in energy or macronutrient intake on the day of aerobic exercise and resistance exercise [24,25]. The effect of resistance exercise on acylated ghrelin levels is less marked and the weight training burns less calories in general. Nevertheless, both endurance- and resistance-trained individuals prefer healthier food choices, which is beneficial for maintaining proper weight [26]. There is limited evidence of suppression or no change in response to PA. At the same time, increases in satiety hormones including PYY, GLP-1 and PP have been reported during aerobic exercise, although, again, these changes appear to be less significant during resistance exercise. However, the hormonal fluctuations are short-lived and usually return to control values in the hours after exercise [27,28,29].

### 3.2. Physical Activity and Sleep

Another pillar of a healthy lifestyle relevant to the development of obesity is sleep. Both sleep disorders—sleep apnea, insomnia and disrupted diurnal rhythms—as well as sleep duration and quality affect cardiovascular factors and the risk of obesity [30].

Short sleep duration can affect carbohydrate metabolism, the hypothalamic–pituitary–adrenal axis and sympathetic nervous system activity. Reduced glucose tolerance and insulin sensitivity result in increased glucose levels and increase the risk of insulin resistance and diabetes. Increased levels of ghrelin and decreased levels of leptin—hormones that regulate feelings of hunger and satiety—result in an increased appetite, causing individuals to take in more energy from food. These changes ultimately correlate with larger waist circumferences and weight gain [31].

In addition, elevated cortisol levels are associated with higher blood pressure. Short sleepers tend to have elevated levels of C-reactive protein and interleukin 6, which correlate with cardiovascular events [32]. Sleeping less than 6 h increases the risk of metabolic syndrome [33] and, in adults, sleep lasting more than 10 h worsens the lipid profile [34].

However, a meta-analysis by Hua et al. did not confirm the negative effect of long sleep duration on the occurrence of metabolic syndrome components [32]. The recommended adequate amount of sleep varies through the lifetime, with more time spent in infancy, childhood and during illness. Ultimately, a sleep duration of 7 to 8 h per night is considered adequate for most of the population of adults. Sufficient sleep is essential for optimal daytime functioning, productivity and well-being, as well as for a reduced risk of disease, including cardiovascular and metabolic diseases [35,36]. Studies show that physical exercise improves subjectively assessed sleep quality, the severity of insomnia and daytime sleepiness. Moreover, regular physical training promotes greater sleep depth and night recovery, especially when performed in the morning. Some studies indicates that sleep could even be disturbed due to the heavy exercises performed in the evenings [37]. The meta-analysis by Xie et al. considered studies that examined the effects of different types of exercise—including aerobic exercise, strength training, walking, pilates and yoga [38]. A study by Sullivan-Bisson et al. that examined the effects of walking on sleep quality and duration showed that the number of minutes of activity per day was positively related to sleep quality, but not sleep duration, which was particularly marked in women [39].

### 3.3. Physical Activity and Psychological Status

Psychological status is also key in terms of overall health. Psychological conditions such as depression, anxiety and psychological stress are associated with chronic metabolic disorders, including primarily central obesity, but also insulin resistance, type 2 diabetes and dyslipidemia [40]. It is worth underlining that the recent COVID-19 pandemic significantly affected psychological well-being and reduced the possibility of performing PA outside due to isolation requirements. Moreover, the long-lasting consequences of the COVID-19 pandemic are still present [41]. PA, due to its strong effect on brain neuroplasticity, positively influences mental health improvement. Neuroplasticity is increasingly being characterized as a central mechanistic component of mental health improvement. At the structural level, neuroplasticity refers to the growth of new neurons and glial cells and new connections forming between existing neuronal networks by remodeling dendrites, creating and pruning synapses, and enlarging axons. Additional changes include an increase in angiogenesis and enhancement of neurotransmitter systems. In adults, neurogenesis is now thought to occur in two specific areas of the brain, both of which are important to mental health disorders: the subangular zone of the dentate gyrus and the subventricular zone adjacent to the caudate nucleus and the striatum (subcortical frontal cortex). The dentate band is essential for the formation and consolidation of new memories and the regulation of affect, changing mainly through the growth of new gray matter cells. The subventricular and caudate areas are crucial for dopamine function in the striatum and subcortical white matter pathways.

Benefits of PA on psychological well-being are well documented. There is no one type of PA (endurance and resistance) or discipline that mostly alleviates psychological disorders. PA should be suited to the individual’s preferences and needs. However, PA reduces the risk of depression, anxiety, chronic stress and several other conditions [42].

To facilitate neuroplastic changes, several key systemic factors must be present, all of which can be modified by physical training. The most important of these are neurotrophins, intact metabolic function of the brain, low levels of neuroinflammation and sleep. Neurotrophic growth factors are enhanced by exercise and linked to neuroplasticity, including modulation of brain-derived neurotrophic factor (BDNF), vascular endothelial growth factor and insulin-like growth factor 1. BDNF increases after aerobic and resistance training and is crucial for neurogenesis in the dentate gyrus [43].

The main hypothesis linking psychological stress to the occurrence of obesity and other metabolic diseases is the dysfunction of the hypothalamic–pituitary–adrenal axis. HPA axis dysfunction raises blood cortisol levels, which increases glucose and insulin levels, leading to insulin resistance, dyslipidemia, high blood pressure and obesity [44]. A meta-analysis indicated that adults exhibiting high levels of stress had a 45% higher risk of metabolic syndrome than adults experiencing less stress [45]. In addition, people with good well-being generally exhibit more health-promoting behaviors—they smoke less often, abuse alcohol less, eat healthier foods, and are more active in their leisure time [46]. Therefore, psychological well-being and psychosocial resources, including life satisfaction and personal development, indicate a reduced risk of obesity and metabolic disease [47].

### 3.4. Physical Activity and Alcohol

A stimulant that particularly increases the risk of obesity is alcohol [48]. This is due to the fact that 1 g of alcohol provides 7.1 kcal (29 kJ) and alcohol is usually consumed in addition to food, hence the increased energy supply and consequent weight gain [49]. In addition, Yeomans points out that alcohol promotes greater food consumption, probably through short-term activation of the reward system [50]. Studies indicate that heavy and binge drinking carries a higher risk of developing obesity [51,52]. Light and moderate drinking, on the other hand, does not significantly increase the risk of obesity [50,52]. PA may promote a reduction in alcohol consumption and be an adjunct to the treatment of alcoholism. However, such an effect of PA has not been demonstrated in the case of binge drinking [53,54]. Finally, it is worth underlining that individuals engaged in regular PA tend to have healthier food choices in general which can explain the beneficial impact of PA on alcohol consumption to some degree [55].

### 3.5. Physical Activity and Social Issues

PA also helps build interpersonal bonds. Undertaking exercise in a group is associated with social support, which can strengthen the identity of the exerciser and increase motivation to continue training [56]. Particularly during the COVID-19 pandemic, PA sharing helped develop a positive personal image and establish new interpersonal relationships [57]. Despite the possibility of introducing lifestyle interventions for patients, specialists point to the complexity of the problem and the significant influence of external factors on the development of obesity. Individuals with lower socioeconomic status have a higher risk of developing metabolic syndrome [58,59]. The treatment of obesity, a major criterion of metabolic syndrome, involves the cooperation of the patient with a multidisciplinary team of specialists, including doctors of various specialties, nutritionists, psychologists and physiotherapists. This approach is justified by the complexity of obesity and the range of lifestyle factors affecting its development. Obstacles to multifaceted assistance are most often psychosocial and economic factors, which require extended systemic measures [58,60]. There are also reduced opportunities to be engaged in PA in low-income countries and isolated societies. For example, a reduced number of public gyms or problems with finding peers to exercise with will negatively impact the willingness for PA [61].

**Figure 1:**
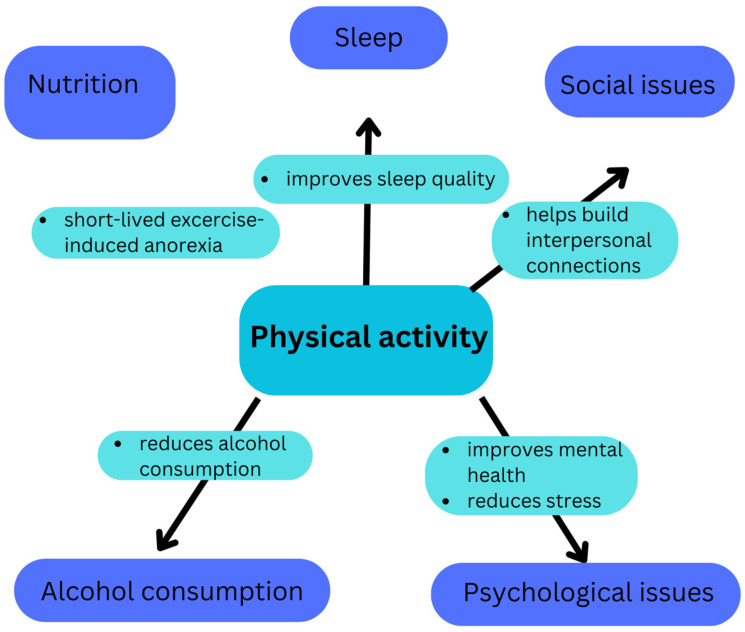
The impact of PA on the pillars of lifestyle medicine.

A summary of the impact of PA on the pillars of lifestyle medicine is presented in Figure 1.

## 4. Types of Physical Activity and Their Impact on Body Composition and Health

Decreased PA is strongly associated with increased obesity [20]. Increasing energy expenditure can help reduce excess body fat [21]. An inverse correlation is observed between PA, body mass index (BMI), waist-to-hip ratio (WHR) and waist circumference. Weight reduction through physical training results in less loss of muscle mass (compared to body fat) than weight loss through diet alone [17,62]. Moreover, given the relationship between lean body mass and resting energy expenditure, higher lean body mass has a protective effect against excessive fat accumulation through greater resting energy expenditure [63]. In addition, a reduction in fat mass promotes increased adiponectin levels and improved cytokine profiles, changes that are associated with metabolic syndrome and the development of insulin resistance. It was observed that a two-year diet and exercise intervention reduced leptin levels and increased adiponectin levels in the subjects. This intervention was also observed to improve glucose control without affecting GLUT-4 gene expression in skeletal muscle in the subjects. In addition, improved insulin sensitivity was associated with improved peak oxygen uptake physical performance [64].

There are two main types of exercise—aerobic and resistance. Aerobic exercise is considered the most effective type of exercise in addressing obesity-related health problems. A study by Bateman et al. found that aerobic training improved metabolic syndrome parameters to a greater extent than resistance training. However, the greatest improvement was seen with a combination of resistance and aerobic exercise [65]. Aerobic exercise can be performed continuously or as HIIT—high-intensity interval training—which is characterized by short intense bursts interspersed with recovery periods [66]. According to the American College of Sports Medicine (ACSM) guidelines, high-impact exercise should be preceded by moderate training to facilitate adherence [67].

Aerobic training causing energy expenditure is an important tool in reducing body weight and body fat, including visceral fat. The best results are obtained when combined with a balanced diet. Intervention with aerobic training alone results in a modest weight loss (0 to 2 kg) and its effectiveness is only possible with high training volumes [68,69]. Aerobic exercise has beneficial systemic effects. The study by Monda et al. checked several blood parameters (aspartate aminotransferase, alanine aminotransferase, gamma-glutamyl transpeptidase and cholesterol (total, HDL and LDL, and triglycerides)) after 6 months of regular aerobic-type PA with no changes in diet. All blood parameters studied improved from baseline levels. An association between increased plasma orexin A levels and PA was also described. This neuropeptide plays an important role in several key states: sleep/wakefulness, eating behavior, mood and energy homeostasis. It has been shown to be involved in adaptations to exercise [70].

There are three main types of PA, which depending on its intensity: light, moderate and vigorous. PA of 1.6–2.9 METs is considered as light and refers to activities such as casual walking and completing household chores; 3–5.9 METs is defined as low and includes brisk walking and low-impact aerobics; and PA ≥ 6 METs, including sport activities, is considered as vigorous [71,72].

Resistance training increases strength, muscle mass and lean body mass to a greater extent than aerobic training. Because of the increase in muscle mass, it does not cause weight loss without restricting energy from the diet. However, even without caloric restriction, it has a beneficial effect on body composition, reducing fat mass, including abdominal fat, and increasing basal metabolism. At rest, skeletal muscle consumes 54.4 kJ/kg (13.0 kcal/kg) per day, more than adipose tissue—18.8 kJ/kg (4.5 kcal/kg) [73]. This is particularly important for people losing weight, as resting metabolism is reduced after weight loss in healthy normal-weight and overweight individuals. This reduction occurs due to the loss of mass of energy-expendable tissues and metabolic adaptations taking place. Consequently, the loss of energy-expendable tissues—mainly skeletal muscle and adipose tissue—contributes to a reduction in resting metabolism [74].

Resistance training has also been shown to increase insulin sensitivity, improve glucose tolerance and lower blood pressure values [73,75].

According to some studies, training at peak fat-oxidation rate, which is known as FAT_max_, helps to burn fat more effectively [76]. Scientific reports on the effectiveness of this type of training are scarce, but a meta-analysis of randomized control trials indicated that it reduces body weight and body fat, and increases cardiovascular fitness in low-physical fitness people with obesity [77].

According to the World Health Organization, adults should exercise weekly at moderate intensity for 150–300 min or at high intensity for 75–150 min. Resistance and endurance training each has its own benefits [78]. However, both types promote different adaptations. Therefore, we underscore the importance of individualized PA recommendations [79].

PA not only includes exercise, but also activities performed outside of training. Attention should be paid to the role of spontaneous non-exercise activity thermogenesis (NEAT). NEAT is crucial for regulating energy expenditure and controlling body weight. It is a highly variable component of daily total energy expenditure, and low levels of NEAT are associated with the incidence of obesity. NEAT levels are highly dependent on individual and environmental factors, including work and leisure time. Activities include going to work/school or completing household chores such as cleaning, cooking or gardening [80].

## 5. Reports on the Impact of Physical Activity on Weight Loss

The effect of exercise interventions in weight loss has been extensively studied—both implemented alone and with concomitant dietary interventions. Below and in Table 1 are details of studies describing the effects of different types of dietary, exercise or combined interventions on weight loss.

In a study on a group of 36 patients, Redman et al. examined the effect of a 25% energy deficit through diet alone or diet and exercise for 6 months on body composition and fat distribution. The results suggested that when the level of imposed caloric restriction is carefully tailored and carefully controlled, changes in body composition and abdominal fat distribution are not further increased by the addition of exercise [81].

The goal of the Donnelly et al. study, on the other hand, was to test whether exercise without energy restriction causes a clinically significant change in body weight in different groups from baseline. They observed that exercise provided significant weight loss (5% on average). The authors noted that supervised exercise at an appropriate intensity resulted in weight loss from exercise alone exceeding that observed in intensive weight loss interventions using a caloric deficit. What is more, weight loss during exercise, while on an ad libitum diet, was entirely due to fat loss [82].

The comparative effects of dietary restriction and increased exercise on weight loss were also studied by Dunn et al. In their observational study on a group of 962 people, it was shown that changes in diet were more effective than increased PA. However, for women, the cumulative effect of simultaneous changes in diet and exercise on weight loss was more marked [83].

The findings from the meta-analysis by Johns et al. indicate that weight loss is similar in the short term for diet alone and combined diet and exercise programs, but weight loss is greater in the long term when diet and PA are combined. Physical activity-only programs are less effective than combined programs in both the short and long term [84].

In contrast, the systematic review found that the most effective regimen for treating obesity in adults is a combination of strength and endurance exercise for at least 175 min per week combined with a hypocaloric diet tailored to the patient’s metabolic needs [85].

In the Elliot et al. study, patients were divided into four groups: dietary intervention only, increased PA only, both and no change. It was observed that participants who increased PA and improved diet were more likely to improve their health than those who implemented only one intervention. In particular, the odds of weight loss were significantly higher in patients who changed both behaviors compared to implementing only one intervention—diet or exercise [86].

Foster-Schubert et al., in their study on a group of postmenopausal overweight and obese women, observed that diet combined with exercise reduced body weight and body fat levels to the greatest extent. In this study, a group of 439 subjects were divided into four groups: diet, exercise, a combination of both and a control group [87].

Similarly, Joseph et al. in a group of obese women aged 40–60 observed that a program that included diet combined with exercise and regular counseling showed the best results in terms of BMI, lean body mass, weight loss, peak oxygen uptake, and body image. Notably, the group combining exercise with diet showed no decrease in lean body mass, unlike the group with dietary intervention alone [88].

These reports indicate that the most effective therapy for reducing body weight without significant loss of lean body mass is a combination of a properly selected diet and physical activity. Diet alone has a negative effect on body composition while training alone does not result in sufficient weight loss.

**Table 1:** Detailed types of PA interventions with their impact on weight loss.

Study	Design and Sample	Intervention	Results
Redman et al. [81]	Design: RCT N = 36 Sample characteristics: 36 overweight but otherwise healthy participants (16 males, 19 females)	C: healthy weight maintenance diet CR: 25% reduction in energy intake, *n* = 12 CR + EX: 12.5% reduction in energy intake + 12.5% increase in exercise energy expenditure L: 6 months Exercise specifics: self-selected three exercise work-loads on a treadmill, stationary cycle or StairMaster	The participants lost 10% of body weight (CR: −8.3 ± 0.8, CR + EX: −8.1 ± 0.8 kg, *p* = 1.00), approximately 24% of fat mass (CR: −5.8 ± 0.6, CR + EX: −6.4 ± 0.6 kg, *p* = 0.99), and 27% of abdominal visceral fat (CR: 0.9 ± 0.2, CR + EX: 0.8 ± 0.2 kg, *p* = 1.00)
Donnelly et al. [82]	Design: RCT N = 141 EX: (*n* = 40); CON: (*n* = 35) Age: 22.6 ± 3.9 years Sample characteristics: overweight and obese participants (body mass index, 31.0 ± 4.6 kg/m^2^)	C: non-exercise control condition; usual ad libitum diets EX: usual ad libitum diets, 400 and 600 kcal/exercise session, primarily walking/jogging on motor-driven treadmills F: 5 days/week L: 10 months	EX: weight loss from baseline to 10 months: 3.9 ± 4.9 kg (4.3%) and 5.2 ± 5.6 kg (5.7%) C: weight gain: 0.5 ± 3.5 kg (0.5%) (*p* < 0.05)
Johns et al. [84]	Design: Systematic review and meta-analysis N = 1022 Age: ≥18 years Sample characteristics: overweight or obese	Group characteristics: EX: moderate to high intensity PA (e.g., brisk walking) 3 to 5 times per week D: energy restriction and recommendation to consume low-fat diet D + EX: combined behavioral weight management programs	from baseline or at 3 to 6 months: no significant difference in weight loss between the D + EX and D (−0.62 kg; 95% CI −1.67 to 0.44) at 12 months: a significantly greater weight-loss in the D + EX (−1.72 kg; 95% CI −2.80 to −0.64) significant weight loss in D + EX at 3 to 6 months (−5.33 kg; 95% CI −7.61 to −3.04) and 12 to 18 months (−6.29 kg; 95% CI −7.33 to −5.25)
Foster-Schubert et al. [87]	Design: RCT N = 439 Sample characteristics: overweight-to-obese postmenopausal sedentary women	Intervention design: D: calorie-reduced, low-fat diet EX: moderate-intensity, facility-based aerobic exercise program D + EX: combination of both interventions C: no lifestyle change L: 1 year	average weight loss at 12 months was −8.5% for the D group (*p* < 0.0001 vs. C) −2.4% for the EX group (*p* = 0.03 vs. C) −10.8% for the EX + D group (*p* < 0.0001 vs. CON) the CON group experienced a non-significant −0.8% decrease BMI, waist circumference, and % body fat were also similarly reduced.
Olateju et al. [85]	Design: Systematic review N = 2191 Age: 18–79 Sample characteristics: adult patients with obesity	Groups characteristics: D: dairy foods alternate-day fasting and calorie restriction Mediterranean diet and Central European diet Companion-intensive multi-aspect weight management and traditional multi-aspect weight management EX + D: combination of intensive PA of about 175 min per week and a portion-controlled diet	dairy-based diet-reduction in body weight (−1.16 kg [−1.66, −0.66 kg], *p* < 0.001) and body fat mass (−1.49 kg [−2.06, −0.92 kg], *p* < 0.001) alternate day fasting: body weight change of mean −0.9% ± 0.6% in the low-weight-loss group, and −9.9% ± 1.1% in the high-weight-loss group caloric restricted weight loss: −1.3% ± 0.7% in the low-weight-loss, and −9.2% ± 1.2% in the high-weight-loss groups. EX + D: more significant weight loss of 5%
Elliot et al. [86]	Design: survey N = 1488 Age: ≥60 Sample characteristics: health program participants	Group characteristics: EX: increased PA, D: changed diet D + EX: increased PA and changed diet CON: no changes to PA and diet	EX: 5.2 times more likely to lose weight in comparison to CON D: 7.2, 2.4 and 3.5 times more likely to lose weight, lower their blood pressure, and lower their cholesterol, respectively, compared to CON D + EX: losing weight much more (17.5) versus EX (5.2) D (7.2)
Joseph et al. [88]	Design: retrospective observational study N = 145 Age: 40–60 Sample characteristics: overweight women	Group characteristics: D: diet protocol with adjustment and customization to each participant with no advice concerning PA EX: training at least 3 times/week, which included: strength training, aerobic training and stretching for 60 min with no diet advice D + EX: customized diet and training protocol same as in EX CON: no intervention L: 8 weeks	D + EX: the best improvements: −1.3 kg/m^2^; body fat, −2.9%; lean body mass, +1.1; VO_2_ max, +4.8; body image D + EX: weight loss of average 3.5–3.9 kg no change in weight loss in EX and CON WHR did not change in either group D: reduction in LBM D + EX: maintained their muscle mass and showed no change in LBM EX: significant reduction only in the WC

N—number; RCT—randomized control trial; PA—physical activity; EX—exercise group; D—diet group; EX + D—combined exercise and diet group; CON—control group; L—length; F—frequency; WHR—waist-to-hip ratio; and LBM—lean body mass.

## 6. Strengths and Limitations

Firstly, we would like to underscore the major strengths of this study. We focused on an important and emerging topic that has several practical implications. We applied a novel and innovative approach where we analyzed the relationship between physical activity and weight loss mitigated by the key factors of a healthy lifestyle. Most often, PA intervention to lose weight does not occur alone, but in combination with other lifestyle modifications. Hence, our article presents a practical point of view. Secondly, we extracted all the exact PA interventions from the analyzed studies and compared them both in the text and in the table. In this way, we minimized the bias in comparison of studies conducted on different populations and under varied protocols.

However, this review had some limitations that should be acknowledged. Specifically, there is no differentiation between gender, fitness level, ethnicity or country of residence in the discussed articles. Secondly, this is a rapidly changing field of science with several research studies being currently investigated. To not overload the text, we also focused on the five most important pillars of a healthy lifestyle. Other pillars should also be analyzed in future studies. Finally, there is a need for regularly updated studies on this emerging topic with a focus on the understudied areas and discussed limitations.

## 7. Conclusions

PA alone does not have a significant weight-loss effect. The greatest benefits are observed when PA is paired with a healthy lifestyle, specifically a proper diet, sleep, mental health, social support and alcohol. More research is needed on the relationship between the weight-loss impact of physical activity, paired with one pillar of a healthy lifestyle alone, and long-term interventions.

## Abbreviations

The following abbreviations are used in this manuscript:

PA	Physical activity
BMI	Body mass index
WHR	Waist-to-hip ratio
NEAT	Non-exercise activity thermogenesis
